# Isolated Human Peripheral Blood Mononuclear Cell (PBMC), a Cost Effective Tool for Predicting Immunosuppressive Effects of Drugs and Xenobiotics

**Published:** 2015

**Authors:** Jalal Pourahmad, Ahmad Salimi


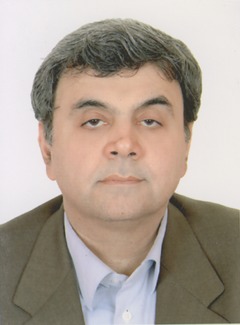


The science of immunotoxicology began in the early 1970s, following the recognition of increased sensitivity to infection following exposure of test species, including monkeys, hamsters, ducks, rats, mice and guinea pigs to various xenobiotics. Reduced resistance to infectious disease was a well-documented consequence of primary and acquired immunodeficiencies, but a novel outcome of xenobiotic exposure, leading some to characterize xenobiotic-induced immunosuppression as “chemical AIDS”. Although the comparison was scientifically inappropriate, “immunotoxicity” was often thought of as synonymous with “immunosuppression” during the formative years of the discipline, although hypersensitivity, allergy, and autoimmunity were recognized as potential exposure outcomes.

Assessment of potential adverse effects on the immune system is an important component of the overall evaluation of toxicity of personal care products, chemicals and drugs. Current public opinion and ethical considerations have stimulated efforts to reduce the number of animals used to test the toxicity of chemicals, drugs and personal care products. However, only limited effort has gone into developing *in-vitro *or *in silico* methods to detect immune dysfunction. This may be at least partially attributable to the sheer complexity of the immune response, although there has been sufficient progress to warrant continued investigation along these lines.

Aperipheral blood mononuclear cell (PBMC) is any blood cell having a round nucleus such as lymphocyte, monocyteor amacrophage. These blood cells are a critical component in theimmune systemto fight infection and adapt to intruders. These cells can be extracted from whole blood using ficoll, a hydrophilic polysaccharide that separates layers of blood, followed by gradient centrifugation, which will separate the blood into a top layer of plasma, followed by a lower layer of PBMCs and then a fraction of polymorphonuclear cells (such as neutronphils and eosinophils) and finally a bottom layer of erythrocytes. The polymorphonuclear cells can be further isolated by lysing the red blood cells. PBMCs are widely used in research and toxicology applications.

Peripheral blood mononuclear cells (PBMC) give selective responses to the immune system and are the major cells in the human body immunity. They contain several types of cells such as lymphocytes,monocytes or macrophages. 

Because peripheral blood is the place whereexposure to chemicals occurs,these fundamentally important PBMCsare prone to be influenced by drugsand chemicals. This is why the availability of PBMCs from peripheral bloodis very important for researchers studying toxicity of new drugs or chemical compounds. There are many Applications for PBMCs in toxicology research including: 

1)New compound toxicity assessment; this is probably the number one reason why researchers need access to PBMCs; it provides answers to questions regarding the toxicity of potential new drug compounds on humans specially on their immune system. Drug toxicity that affects PBMCs can cause a variety of serious, even life-threatening, toxic side effects, including suppression and toxicity of immune system. PBMCs are also critical tools for predictive studies determining the dosage limit of new drug compounds.

2) Side-by-side comparison studies; researchers typically need both normal PBMCs and diseased PBMCs to compare in side-by-side studies, to get to the molecular heart of a specific toxic response. Understanding which pathways or molecules are impacted helps drug research proceed more rapidly, with greater efficaciousness and low immuntoxicity. 

3)Occupational exposure research; Job and/or environmental exposures to toxic compounds such as heavy metals or benzene can have extremely deleterious effects on immune system. For example, benzene is known to affect stem and progenitor cells in bone marrow.Research has shown that immunotoxicity occurs in people exposed to significant levels of benzene. 

4) Chemotherapy toxicity impact studies; Studies of patients suffering from non-Hodgkin’s lymphoma, breast cancer and non-small cell lung cancer (NSCLC) have concluded that older individuals tend to exhibit greater toxicity after receiving cytotoxic chemotherapy compounds, as compared to younger patients. Studies have continued to explore this causal association between age and PBMCs toxicity. The study concluded that, contrary to prevailing views, olderNSCLC patients do not show a greater PBMCs suppression when compared to younger patients after chemotherapy treatment for NSCLC.

5) Personalized medicine/pharmacogenomics; as the field of individualized drug therapy advances, a better understanding of the “why” behind individual variation in drug response becomes increasingly important.Recent studiesfocus on the genetic variations of drug target, metabolism, transport, and safety. The ultimate goal of such studies is to be able to introduce new medications that are both safe and effective for specific genetic profiles. Because PBMCs are common targets for toxic reactions, it is an important tool for researchers exploring the new and exciting horizons of pharmacogenomics.

*Jalal Pourahmad is currently working as full professor at the Department of Pharmacology and Toxicology, School of Pharmacy, Shahid Beheshti University of Medical Sciences, Tehran, Iran. He could be reached at the following e-mail address: j.pourahmadjaktaji@utoronto.ca*

